# Access to In-Network Hospitals in Tennessee During the COVID-19 Pandemic

**DOI:** 10.1001/jamahealthforum.2022.0063

**Published:** 2022-03-11

**Authors:** John A. Graves, Khrysta Baig, Melinda Buntin

**Affiliations:** 1 Department of Health Policy, Vanderbilt University School of Medicine, Nashville, Tennessee; 2 Deputy Editor, *JAMA Health Forum*

## Abstract

This cross-sectional study assesses the daily percentage of floor and intensive care unit bed available at in-network hospitals for patients with COVID-19 in Tennessee.

## Introduction

Throughout the COVID-19 pandemic, surges in hospitalized patients have resulted in deferrals of nonemergency care, ambulance diversions, and the inability to accept patients in transfer. Private health plans typically require that patients seek treatment at an in-network hospital to minimize out-of-pocket costs. Yet while most patients with COVID-19 were shielded from the financial consequences of treatment at an out-of-network facility until early 2021,^1^ this policy was not the case during the subsequent Delta and Omicron waves, nor was it ever the case for patients with non–COVID-19 conditions. To understand how COVID-19 affected access to in-network care for patients, we paired daily data on the staffed bed capacity in Tennessee with insurance network data to assess the daily percentage of floor and intensive care unit (ICU) beds available at in-network hospitals.

## Methods

We used 2021 data on hospital networks for Medicare Advantage, small-group employer, and individually purchased (ie, Marketplace) plans available in Tennessee from Vericred, an analytics firm that obtains provider directory (ie, a list of in-network clinicians and facilities) information directly from insurers and from online plan directories.^1,2^ We paired these data with information from the Healthcare Resource Tracking System,^2,3^ which collected harmonized data feeds on capacity, including available floor and ICU beds, from nearly all Tennessee hospitals from April 1, 2020, through December 27, 2021.

In this repeated cross-sectional study, we calculated the average daily percentage of available in-network floor and ICU beds. We calculated this percentage separately for each network and then plotted the daily average for each insurance market segment. This study followed the Strengthening the Reporting of Observational Studies in Epidemiology (STROBE) reporting guidelines, and was approved by institutional review boards at Vanderbilt University Medical Center and the Tennessee Department of Health.

## Results

**Figure 1.**
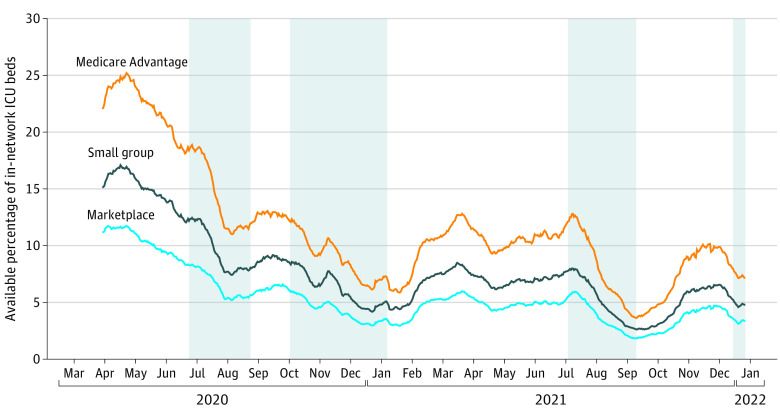
Percentage of In-Network Intensive Care Unit (ICU) Beds in Tennessee by Day and Insurance Network Type

**Figure 2.**
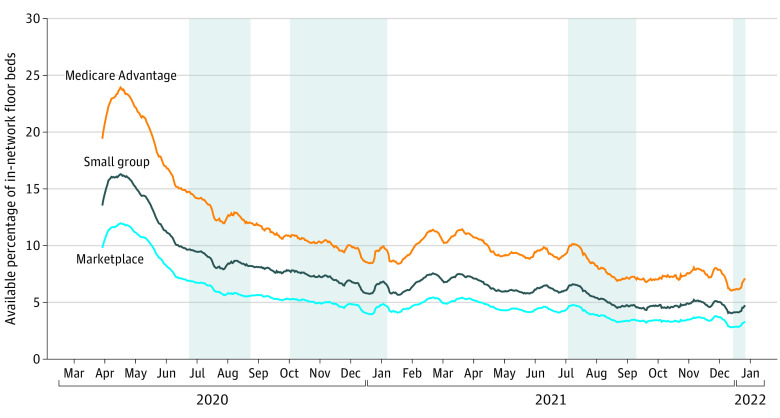
Percentage of In-Network Floor Beds in Tennessee by Day and Insurance Network Type

In total, we analyzed daily capacity data for hospitals within 31 networks (7 Medicare Advantage [22.6%], 16 small group [52.6%], and 8 Marketplace [25.8%]). As the pandemic reached the US in March 2020, in-network floor bed availability was 19%, 14%, and 10% for Medicare Advantage, small-group employer, and Marketplace plans, respectively (Figure 1). The availability of in-network beds declined during each major wave. During the Delta and Omicron surges, floor bed availability reached its lowest points at 6%, 4%, and 3% for Medicare Advantage, small-group employer, and Marketplace plans, respectively. In-network ICU bed availability also declined, with relatively high availability during Spring 2020 at 22%, 15%, and 11% for Medicare Advantage, small-group employer, and Marketplace plans, respectively (Figure 2). During the Delta wave, ICU bed availability declined rapidly, to 4% for Medicare Advantage plan networks and only 2% for Marketplace plan networks. Because of the lag between COVID-19 infection and the need for critical care, the full effect of the Omicron wave on ICU capacity has not yet been experienced, but as of December 27, 2021, in-network ICU capacity was less than 10% for all network types, leaving little room to absorb the demand for ICU capacity seen during previous waves.

## Discussion

This cross-sectional study found broad declines in the availability of in-network hospital beds during the COVID-19 pandemic. Limitations included possible inaccuracies in directory data and unreported daily shifts in bed availability. Nevertheless, the results are significant because patient financial protections for COVID-19 care had mostly fallen away by early 2021 and never protected patients who needed care for conditions other than COVID-19. Additionally, even when a few beds are available, hospitals may choose to not accept ICU transfers to maintain ICU capacity for the escalation of their already admitted patients. Given that medical bills are the largest source of bills in collections,^4^ and that 1 in 6 households report having insufficient savings to cover an emergency $1000 medical bill,^5^ the lack of in-network bed availability has the potential to exacerbate financial challenges that patients face. Moving forward, pandemic planning should include consideration of the financial consequences of limited in-network hospital bed availability during surges. These efforts could include temporary extensions of cost-sharing waivers for out-of-network charges for all patients, or expansion of the Affordable Care Act’s caps on patient out-of-pocket spending to include out-of-network services.
